# Sensing spatial inequality of socio-economic factors for deploying permanent deacons in the UK

**DOI:** 10.3389/fsoc.2024.1481413

**Published:** 2024-11-01

**Authors:** Md. Tariqul Islam, Paul Rooney, Peter McGrail, Sujit Kumar Sikder, Mark Charlesworth

**Affiliations:** ^1^Department of Geography and Environmental Science, Liverpool Hope University, Liverpool, United Kingdom; ^2^School of Water, Energy and Environment, Cranfield University, College Road, Cranfield, United Kingdom; ^3^School of Natural Sciences, College of Health and Sciences, University of Lincoln, Brayford Pool, Lincoln, United Kingdom; ^4^Department of Theology, Philosophy and Religious Studies, Liverpool Hope University, Liverpool, United Kingdom; ^5^Leibniz Institute of Ecological Urban and Regional Development, Dresden, Germany

**Keywords:** sustainable development, open dataset, spatial analysis, hot spot analysis, network analysis, multicriteria assessment

## Abstract

Integrating spatial inequality perspectives in strategic decision-making can ensure positive impacts on resource distribution for public welfare and sustainable development. This study aims to apply evidence-based approaches in deploying permanent deacons. The empirical case study has been conducted at the St Helens denary of the Liverpool archdiocese, UK. Assisting with charitable works is one of three served areas by the Roman Catholic Church facilitated by deacons. The deployment of permanent deacons could benefit from being evidence-based so that a deacon can serve to ease the socio-economic (e.g., population density, long-term health conditions, housing system, employment status, education level, social status) inequality in the most deprived area. We used geographic information system (GIS) based algorithms, Getis-Ord Gi* for hot spot analysis to find the clustered area by considering the socio-economic factors. The open/freely available government census dataset was found to help extract socio-economic parameters. Furthermore, a GIS-based multi-criteria assessment technique was conducted by applying map algebra (raster calculator) to identify the deprived area with ranks considering multiple socio-economic conditions, where served areas by the existing deacons were considered to constrain. The served areas were estimated by applying network analysis where OpenStreetMap and location existing deacons were used as input. Our empirical case study identified the central and northern parts of the deanery as the most and least deprived areas, respectively. Finally, Liverpool archdiocese could consider deploying new permanent deacons in St Helens denary based on suggested deprivation ranks. Therefore, the appropriate number of deacons in the deprived areas can quickly and effectively respond to the needy and enhance communities’ resilience and sustainable development by ensuring proportionate resource distribution.

## 1 Introduction

Spatial inequality refers to the inequality distributions in terms of various geographic regions. Socio-spatial inequalities often map spatial phenomena regarding resources, opportunities, and outcomes. The word ‘inequality’ is often method interchangeable in literature “disparity”; more recently, similar topics are discussed by mentioning “equity” (e.g., Todes and Turok, 2018; de Castro Mazarro et al., 2022; Sikder et al., 2023). Apart from the underlying debate in academic literature, understanding these inequalities is crucial for policymakers in public policy evaluation as they seek to develop effective strategies and actions to address the underlying causes and reduce their adverse impacts (Talen et al., 2018). Recent research offers valuable findings that can inform policy development in this context. Socio-spatial inequalities are closely connected to differences in income and wealth. The concentration of poverty and wealth in specific areas underscores the importance of policies encouraging inclusive economic growth, ensuring access to quality education, and implementing targeted social support programs (Zhang et al., 2021). Engaging communities and stakeholders in decision-making is crucial for effectively addressing socio-spatial disparities. Policymakers should prioritize participatory approaches, empowering marginalized groups and involving them in shaping policies that directly affect their lives (Falanga and Nunes, 2021). Above all, recent research underscores the multifaceted nature of socio-spatial disparities and provides valuable insights for policymakers. Decission-making in the deployment of permanent deacons in catholic communities is such an issue that needs to consider socio-spatial inequalities (or equalities) for better serving the communities.

The word “Deacons” came from the Greek word “Diakonos,” meaning servant. They are called to serve the people of God (Diocese of Salford-DoS, 2023). Men ordained as deacon by the Bishop of his diocese to serve the Church through (i) the Ministry of the Altar, (ii) the Ministry of the Word, and (iii) the Ministry of Charity (Diocese of Salford-DoS, 2023; Diocese of Westminster-DoW, 2023). Deacons are also involved in other ministries, e.g., school, prison, and hospital chaplaincy at the parish level. A deacon can be (a) transitional, “usually seminarians in the last stage of training before being ordained into the priesthood,” or (b) permanent, “ordained into the diaconate to remain in that role” (Diocese of Salford-DoS, 2023). Permanent deacons support their parish priest, engage in the life of their parish, and work to serve and exercise some leadership roles within the community (Diocese of Westminster-DoW, 2023). So, a permanent deacon must actively serve the poor and needy people at the community level through charity. Therefore, a permanent deacon’s deployment must be need-oriented and evidence-based, following the local community.

**Figure 1:**
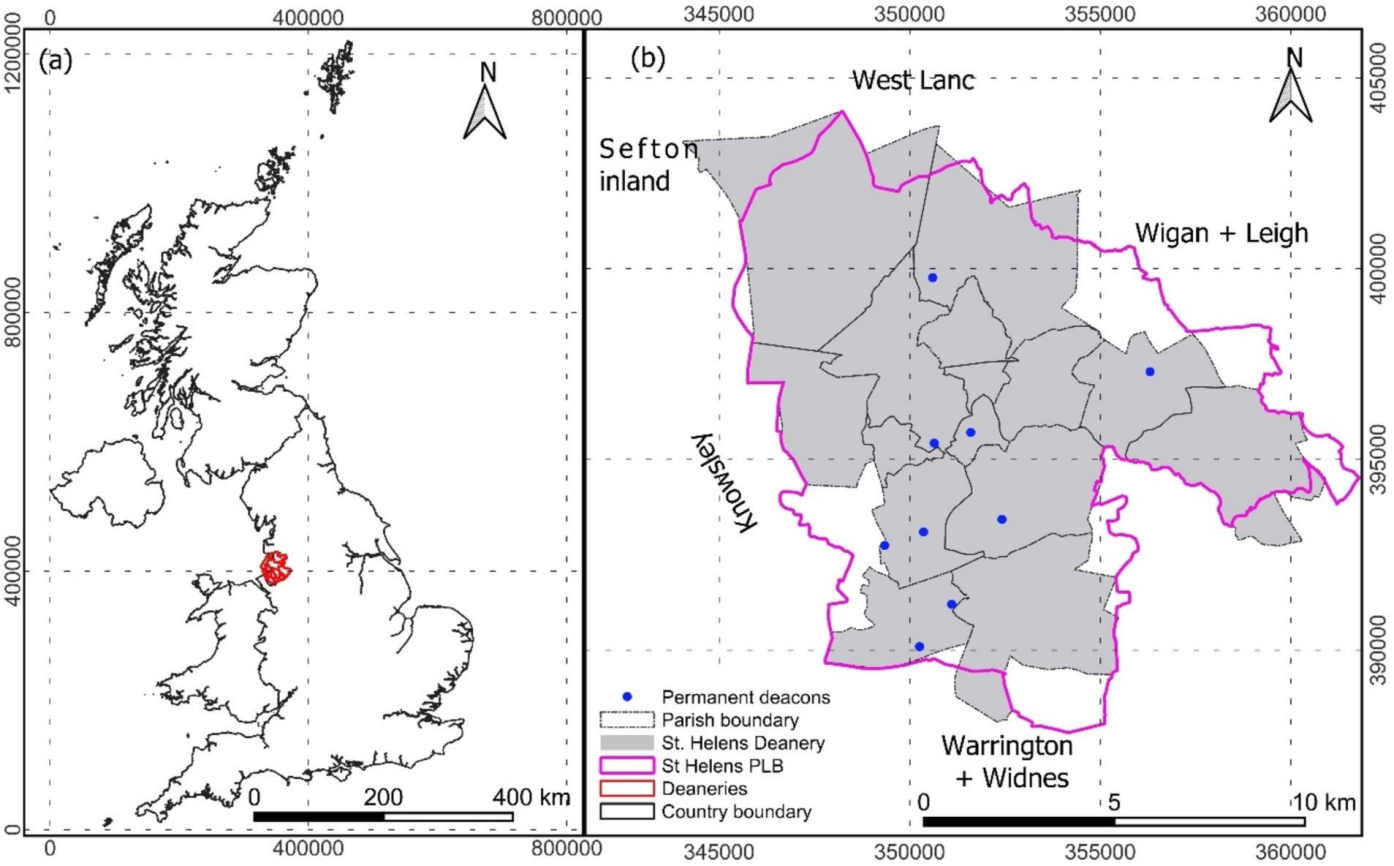
Study area; country layout (a) and St. Helens (b) with the including parish boundary of St Helens deanery and St Helen political boundary (PLB) (the council boundary).

Theoretically, a permanent deacon is assigned to the parish and tasked by the archbishop, considering the diocese’s needs (Diocese of Salford-DoS, 2023). Practically, that might not be the case. The deployment of permanent deacons in St Helens Deanery (Figure 1) in the Archdiocese of Liverpool is investigated to examine such a statement. Spatial inequalities of socio-economic conditions are studied to understand the rational deployment of permanent deacons in St Helens Deanery.

Geographic Information System (GIS) is widely used to investigate spatial inequalities of socio-economic factors (e.g., de Castro Mazarro et al., 2022; Kadi et al., 2022; Lynam et al., 2023; Roy et al., 2024c, 2024b, 2024d). However, the application of GIS to investigate spatial inequalities in the allocation religion religion-related services is unique. There were no such studies found in our literature search boundaries and accessibilities. Therefore, this research provides a unique methodological contribution to the existing literature on religious sectors as a service provider. This research is a two-fold study. Our first objective is (i) to identify socio-economic inequalities of St Helens Deanery on a spatial scale by applying GIS. Based on the results of objective (i), a rational location of deployment of permanent deacons in the deanery is proposed by applying a multi-criteria decision-making tool using GIS (e.g., Ng’ethe and Jalilinasrabady, 2023; Önden et al., 2023), a second objective (ii).

## 2 Study area

St Helens Borough Council (SHBC) (St Helens political boundary in Figure 1) is about 13,600 ha [hectares] and is home to over 180,000 people in 2021, is 4.5% higher than in 2011, with 4,800 businesses and has a strong identity and cultural history linked with industrial heritage (Office for National Statistics, 2021; St Helens Borough Council-SHBC, 2022). 49.2% of the population of St Helens are male, and the remaining 50.8% are female, where 18% are children aged <16 years and the rest of 61 and 21% population are working age group 16–64 years and elderly 64+ years, respectively (St Helens Borough Council-SHBC, 2022). Ethnically, St Helens is less diverse as 98% of residents claim to be White British than England’s average, e.g., 85% (St Helens Borough Council-SHBC, 2022).

The multiple deprivation index in 2019 suggests that St Helens is England’s 26th (out of 317) most deprived local authority. Almost one-fourth of St Helens inhabitants live in the country’s 10% most disadvantaged neighborhoods. The children (<16 years) are affected the most in the central part of St Helens due to income deprivation. About 22% of the people in St Helens are economically inactive. In the education and skills domain, St Helens is the country’s 72nd most deprived authority (out of 317), with the most deprived areas in the central city. Life expectancy for males and females in this area is 77.5 years (79.4 years in England) and 81.0 years (83.1 years in England), respectively (St Helens Borough Council-SHBC, 2022; St Helens Public Health Team, 2020). Again, the highest 20% (first quantile) of vulnerable people live in the central part of the city (St Helens Public Health Team, 2020).

However, St Helens, as a Deanery of Liverpool Archdiocese (Roman Catholic Faith), has a slightly different geographical shape and aerial coverage of about 12,475 ha (Figure 1). It consists of 15 Parishes. Nine deacons live and serve the deanery. Two parishes have four permanent deacons (two for each), and five parishes have five permanent deacons (one for each). The rest of the eight parishes have no deacons (Figure 1), which might be because of limited resources and an inappropriate process of deployment.

## 3 Methodology

### 3.1 Data

**Figure 2:**
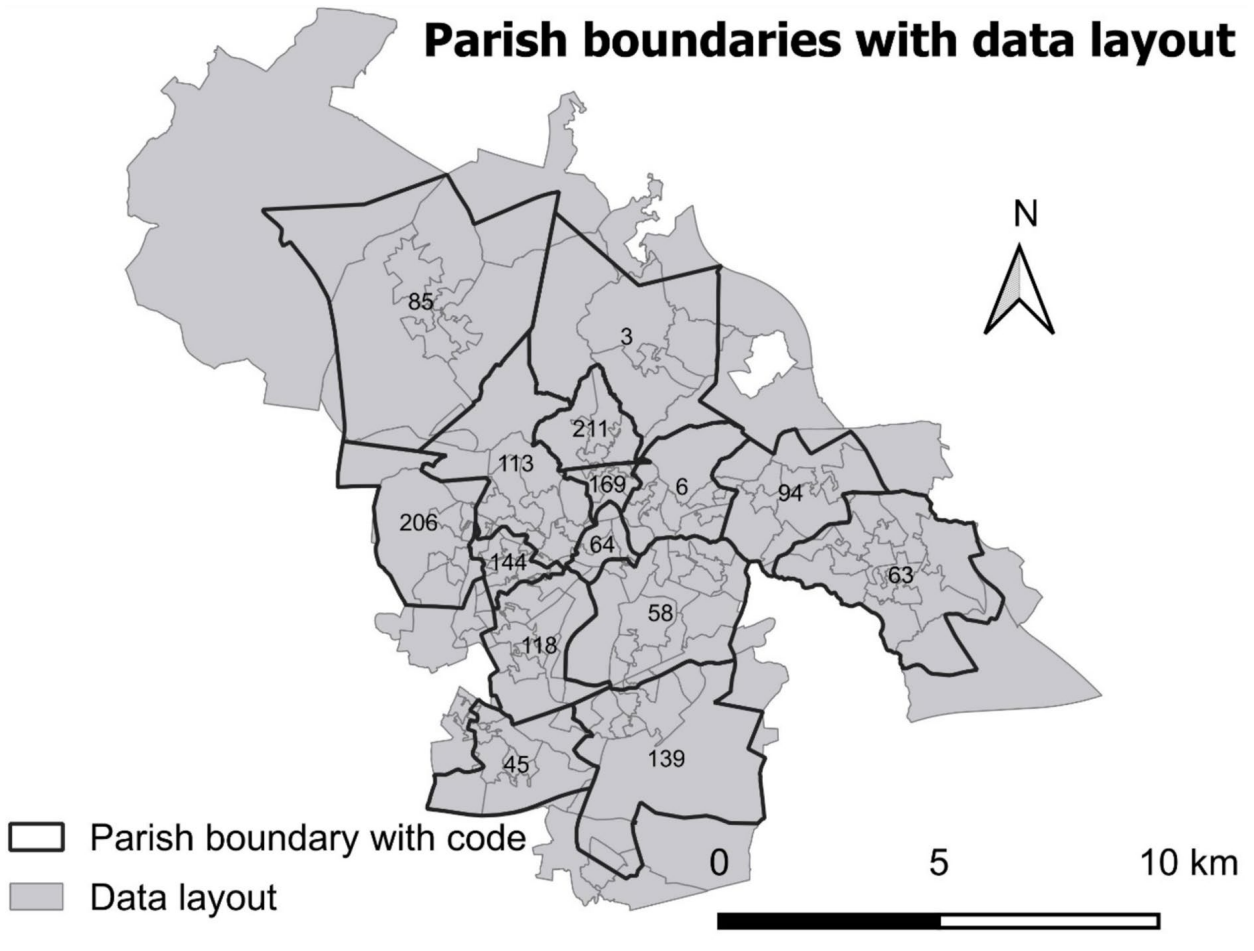
Boundary problem between statistics vs. Parish boundaries.

Socio-spatial inequality is a multifaceted phenomenon affecting personal activities across various fields, even though many studies heavily depend on individual and household income levels. However, this study investigates slightly different angles to socio-economic parameters other than income listed in Table 1. The choice of these parameters is based on publicly available accessible data sources. This is the UK Ordnance Survey 2011 data available at Digimap which is operated by the University of Edinburgh (Digimap, 2024). The data was occupied in Geopackage format with a scale 1:2500 using a rectangle mask that could cover the study extent as in Figure 2. The Geopackage formatted data were accessed to ESRI’s product ArcMap 10.8.1 version and saved to polygon shape format with reference system EPSG: 27700-OSGB 1936/ British National Grid. Ordnance Survey 2021 data was the preference but is still not publicly available on a detailed scale as necessary for this study.

**Table 1:** Socio-economic parameters used in this study.

Ids	Categories	Variables
1	Long term health condition	Category 1: limited a lot
2		Category 2: limited a little
3	Employment	Unemployment
4		Retired
5		Students
6	House ownership	Owned
7		Rental
8	Qualification	Education levels 1–3 and work-related education
9		Education levels 4 and above
10		No qualification
11	Social status	Grade 1
12		Grade 2
13		Grade 3
14		Grade 4

#### 3.1.1 Long-term health condition

According to the (Economic and Social Research Council, 2024), a long-term health condition in the UK is defined as a health problem or disability of a person that limits day-to-day activities for at least 12 months or is expected to be affected for 12 months. It is classified into three groups with details of a long-term health problem or disability with day-to-day activities (a) limited a lot (category 1), (b) limited a little (category 2), and (c) activities not limited. Since the long-term health condition does not limit day-to-day activities, the third category is ignored in this study.

#### 3.1.2 Employment

Unemployment, retirement, and students are considered in this study. The UK adopted the definition of unemployment for International Labor Organizations as “anybody who is without work, available for work and seeking work is unemployment.” The UK considers unemployed who have actively sought work in the last 4 weeks and are available to join work in the next 2 weeks. Therefore, those are students who are studying full-time or part-time but not seeking work. Aged people over 65 years old are considered to be retired (Office for National Statistics, 2024).

#### 3.1.3 House ownership

House ownership refers to the house’s occupancy, whether owned or rented. The rented house includes a social, private, and other rented house. Therefore, a unit of house ownership is a household.

#### 3.1.4 Education

Three levels of education/qualifications of persons are considered in this study. Levels 1–3 and others with education from O to A levels and vocational and work-related qualifications are considered. Level 4 is considered from a bachelor’s degree to higher (e.g., Master’s, Doctorate). And no qualification indicates someone with no academic or professional education.

#### 3.1.5 Social grade

Social grade in the UK is categorized based on the working type and status of household-reference persons aged 16–64 (UK Geographics, n.d.). Therefore, all household members bear the same social class (IPSOS, 2009). According to this definition, Grade 1: higher & intermediate managerial, administrative, professional occupations; Grade 2: supervisory, junior managerial, and administrative professional occupations; Grade 3: skilled manual occupations; and Grade 4: semi-skilled unskilled occupations, lower grade occupations, and unemployed.

Besides, total and age-specific populations were also taken from Ordnance Survey 2011 from Digimap. The parish boundary of St Helens Denary of Liverpool Archdiocese was collected from the Department of Geography and Environmental Science, Liverpool Hope University, Liverpool, UK.

### 3.2 Data harmonization

The layout of downloaded data from Digimap is not similar to Parish boundaries. Data layout follows civil boundaries. Within a parish boundary, several civil boundaries (geographical units) exist. Some of them cross a parish boundary and are shared by different parishes (Figure 2). There are 123 geographical units in the data layout (gray polygon boundary in Figure 2) with a total area of 19691.6 ha. The density of each geographical unit in the civil boundary was calculated with the unit person/ha (gray polygon boundary in Figure 2). The calculated minimum and maximum size of the unit were 14.1 ha (density 102.8 person/ha) and 3725.12 ha (density 0.5 person/ha), respectively, with an average size of 160.1 ha and a standard deviation of 391.16. Then the density layer was clipped based on Parish boundaries. Parish-specific total population and population density (person/ha) using a clipped layer were calculated.

### 3.3 Hot spot analysis

Hot spot analysis performs spatial clustering. Getis-Ord Gi* statistics is widely applied for spatial clustering (e.g., Braithwaite and Li, 2007; Jana and Sar, 2016; Hassan et al., 2022; Roy et al., 2024c) to investigate spatial inequalities over the alternatives, e.g., Geary’s C, Moran’s I, Kernel density (e.g., Majumder et al., 2023; Roy et al., 2024d, 2024a, 2024b). It detects hot spots-local pockets of dependence in data and for comparative advantage of the application of Getis-Ord Gi*, readers are encouraged to read Braithwaite and Li (2007). It generates Gi* index from −3 to 3 where ±3, ±2, and ± 1 reflect statistical significance with a 99, 95, and 90% confidence level, respectively, and 0 is not statistically significant. The negative value indicates cold, and the positive value indicates hot cluster. The spatial clustering, shown by Gi* is controlled by *z*-score and *p*-value. A *z*-score near “0” gives no apparent spatial clustering. In contrast, a high z-score and small p-value indicate a spatial cluster of high values, and a low negative *z*-score and small *p*-value indicate a spatial cluster of low values (Amiri et al., 2021; ESRI, 2023). For more details about *z*-score, *p*-value, confidence level, and Gi* formulation, readers are encouraged to read Amiri et al. (2021), ESRI (2023) and Roy et al. (2024c).

Hot spot analysis was conducted by applying ESRI’s product ArcMap 10.8.1 version. Getis-Ord Gi* is available under ArcToolbox/Spatial Statistics Tools/Mapping Clusters. The parameters were set as K Nearest Neighbors to Conceptualization of Spatial Relationships, Euclidean Distance to Distance Method, and ROW to Standardization, which composition widely applied in scientific communities (e.g., Sikder et al., 2019; Amiri et al., 2021). To test the sensitivity of the number of neighbors in clustering, we applied 4–12 to the Number of Neighbors using long-term health conditions limited with a lot (Appendix Figures A1, A2). The results were given to Cold spot - 99% con. (−3), Cold spot - 95% con. (−2), Cold spot - 90% con. (−1), Not significant (0), Hot spot - 90% con. (1), Hot spot - 95% con. (2), and Hot spot - 99% con. (3). Increasing the number of neighbors shifts from no influence to either cold or hot spots (Appendix Figure A2). These results were compared with reports from the local council authority (St Helens Public Health Team, 2020; St Helens Borough Council-SHBC, 2022). The number of Neighbors to 8 provided the best matching. Therefore, all Getis-Ord Gi* models were run by applying 8 to the Number of Neighbors. Spatial clustering was applied to the data layout as specified in Figure 2. Further, hot spot resultant layers were clipped based on parish-level data specified in Figure 2 for visualization and further analysis, e.g., multi-criteria assessment in the next section. This gave no integration error with the parish-level population and density layers which were prepared in section 3.2.

### 3.4 Multi-criteria assessment approach

Aggregation is conducted by executing Multi-Criteria Assessment (MCA) (section 3.4.5) (Heywood et al., 2011, p. 244). The MCA allows combining multiple sets of criteria and imposing weight to reflect the relative importance of those criteria [readers are encouraged to read (Heywood et al., 2011, p. 244–247) to understand more details about MCA]. Criteria for MCA were identified by the outcome of the structured questionnaire survey and experts’ opinions.

#### 3.4.1 Questionnaire survey and selection criteria

A structured questionnaire survey was conducted online. The questionnaire was prepared on a Likert scale (Sullivan and Artino, 2013), a 5-point ordinal scale where 1 and 5 indicate strongly disagreed and agreed, respectively and 3 indicates somewhat agreed (a neutral point) (Appendix B1). Before the survey, ethical approval was taken from the Ethics Committee of Liverpool Hope University, Liverpool, UK. Before, sending the questionnaire, a pilot testing was conducted with a permanent deacon and a priest in the St Helens Deanery. Based on the pilot test, a modification was conducted and finally, it was sent to the respondents.

The primary respondent was Rev. Christopher A Fallon, Chair of the Ministry Research Project, Archdiocese of Liverpool, Liverpool, UK, who is fully identifiable. Besides, a priest and a permanent deacon (in the St Helens area) completed the survey, who are fully anonymous. The survey was conducted with four key persons (including an academic) who were fully aware of the role of a permanent deacon in the Roman Catholic community and had the best local knowledge. The questionnaires were sent, and the response sheets were received through email. Responses were processed in Microsoft Excel. Each response from the respondents was arithmetic averaged (the sum of the weight of each factor was divided by the number of respondents, in our case the total number of respondents was four). Since the respondents were limited and weighting scores were bounded by 1 to 5, we considered all responses.

**Figure 3:**
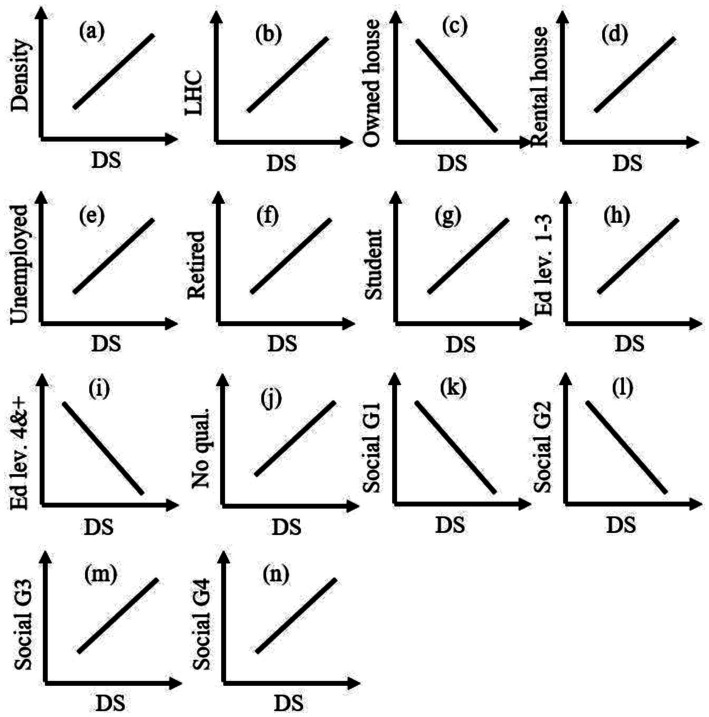
Relationship of deacons/deacons’ services (DS) with (a) density, (b) long-term health condition (LHC), (c) people with owned house, (d) people with rental house, (e) unemployed, (f) retired, (g) students, qualification with (h) level 1–3 and work-related education, (i) level 4 and above, and (j) no education, (k) social Grade 1, (l) social Grade 2, (m) social Grade 3, and (n) social Grade 4.

Criteria are categorized into (i) factor and (ii) constraint. Factors have some degree of influence on decision-making. This influence could be positive or negative, as in Figure 3. When the average value (from the questionnaire survey) is >3, it means the need for deacons or deacons’ services is positively influenced. However, when it is <=3, it means the demand for services is negatively influenced (Figure 3). In such a manner, factors are identified (listed in Table 2). And the constraint is identified to the served area by the existing deacons.

**Table 2:** Relative weight derived from the questionnaire survey.

Factors	Categories	Weight value
Density		4.50
Long-term health condition	Limited a lot	4.25
	Limited a little	4.25
Home ownership	Owned home	1.75
	Rental home	4.25
Economic	Retired	3.50
	Students	3.50
	Unemployed	4.75
Qualification	Education level 1–3 and work-related	3.50
	Education level 4 and above	2.25
	No qualification	4.25
Social status	Grade 1	2.50
	Grade 2	3.00
	Grade 3	3.50
	Grade 4	4.25

#### 3.4.2 Data standardization

Factors were different in scale, e.g., population density and long-term health condition. To facilitate the comparison of factors, they should be in the same unit and scale (Heywood et al., 2011, p. 245). The scale of output of hot spots analysis was 7 (−3, the coldest, to +3, the hottest), which was rescaled to 1 (coldest) to 7 (hottest). This scaling was chosen because it preserved the originality of hot spot results, but values were changed linearly. The areas with values 1 and 7, e.g., for long-term health conditions and unemployed people, indicate that these areas need deacons’ services the least and the most, respectively. On the other hand, areas dominated by the owned housing system indicate other ways around (negatively influence, see Figure 3), areas with value 1 (coldest) need more deacons/deacons’ services. Therefore, the value was inverted for all factors (e.g., owned house, education levels 4 and above, and social grade 1) that had a negative influence (Figure 3).

To conduct the rescaling of the factor, the data layers (output of hot spot analysis) were converted from vector to raster. Spatial resolution was set to one m^2^. The density layer was converted to a raster with seven classes following a quantile classification scheme where 1 and 7 indicate the least and the densest area.

#### 3.4.3 Identification of key constrain

Besides the questionnaire survey, the respondents were asked, “usually, how far a deacon serves.” The area served by the existing deacons is considered to be constrained. A deacon’s travel distance estimates the served area. An area of 1 km distance (in experts’ opinion) from the existing deacon’s location is considered to serve the area. However, in this case, 1 km, one and a half km, and 2 km travel distance from the existing deacons’ location were considered to make a scenario map. The served area was considered a null (0) value and the rest of the study area was set to 1, facilitating the Boolean operators in raster overlay (Heywood et al., 2011, p. 195).

**Figure 4:**
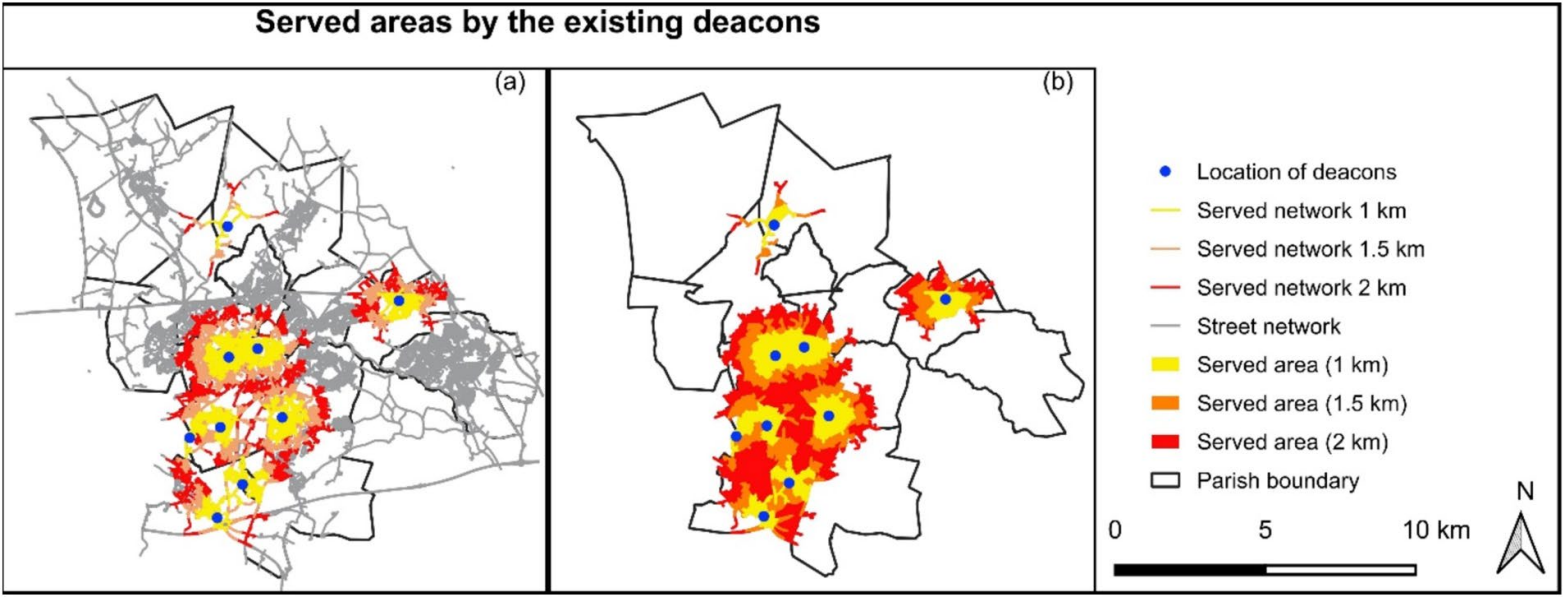
Served road network (a) and area (b) by the existing deacons.

To estimate the served area, road network data from OSM (OpenStreetMap) was used (OpenStreetMap Foundation, 2018). Data was downloaded and analyzed using QGIS 3.16 (QGIS, 2021). Service area (from layer) of Network analysis in QGIS 3.16 was used to estimate the served area (Figure 4). The OSM layer was set to a vector layer representing the network, location of permanent deacons (point layer) was set for starting points, and 1 km, 1.5 km, and 2 km was set to travel cost (distance). In this way, the server network was identified. A buffer zone of 50 m from and around the network was created to identify the served area (Figure 4).

#### 3.4.4 Expert-based weights allocation

The need for deacons and deacons’ services is influenced more by one factor than others (e.g., unemployed people need more deacons’ services than retired people). Weight reflects the relative importance of factors (Heywood et al., 2011, p. 246). Each factor is multiplied by the corresponding weight obtained from the questionnaire survey (Table 2).

#### 3.4.5 Multi-criteria assessment

Multi-criteria assessment is executed by applying a raster calculated in QGIS 3.16 following the equation below (e.g., Jain and Subbaiah, 2007; Topuz and Deniz, 2023):


$$MCA=C\sum_{k=1}^{n}f^{k}w^{k}$$

where, *C* is the constraint (served area by existing deacons), *k*^th^ is the number of a specific factor (*f*) (rescaled hot-spot output), *w* is the corresponding weight (obtained from the expert survey in Table 2), and *n* is the total number of factors. In this case *n* = 15.

Aggregated layers (output from MCA analysis) were classified into five classes using a quantile approach where one indicated the least and five indicated the most deprived area for deacons and deacons’ services.

#### 3.4.6 Validation

**Figure 5:**
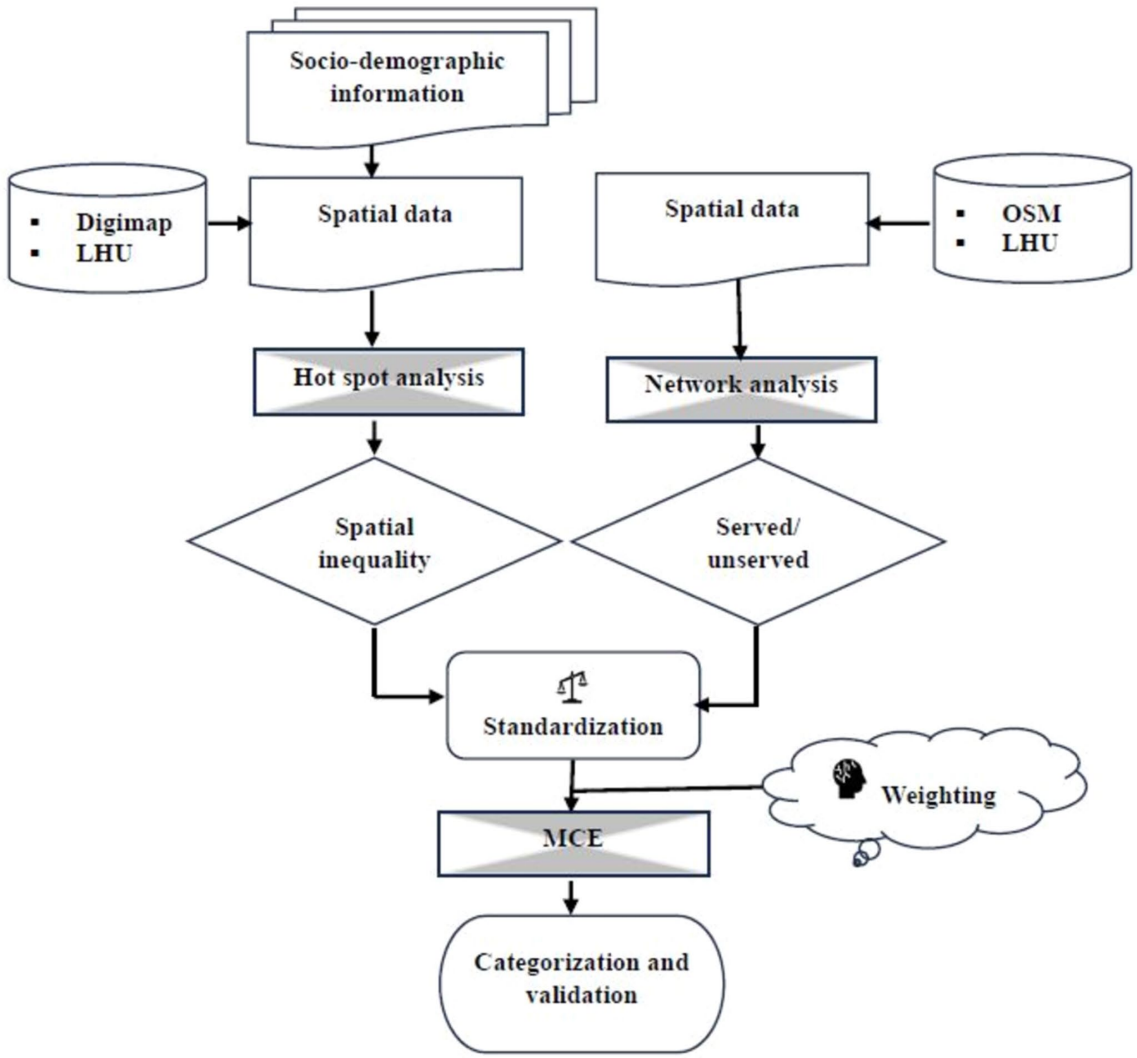
Methodological flow diagram.

The outcomes from this study were presented in a workshop organized by the Archdiocese of Liverpool, UK. The internal and external stakeholders were present there. The local stakeholders compared the results with the local situation. Further, the results and process of this study were presented at an international conference (Sixteenth Annual Conference of European Academy of Religion, 19–23 June, St Andrews, UK).^1^ Scholarly comments to validate the process of this study were documented and analyzed to understand the applicability of these methodologies in religious service/resource allocation. The comments were analyzed in narrative format (Islam et al., 2021, 2022). Note that no matrices were used to quantify or to identify the sensitivity. Methodological flow is shown by the diagram in Figure 5.

## 4 Results

### 4.1 Population and density

**Figure 6:**
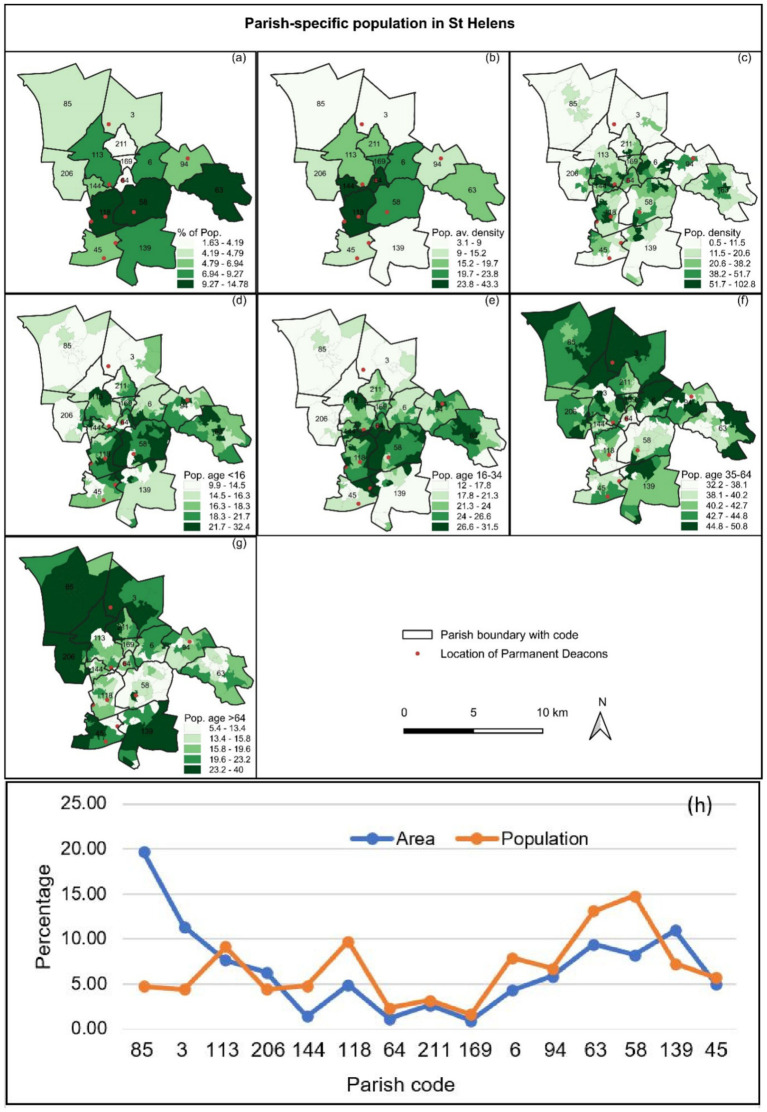
Location of permanent deacons with (a) parish-specific percent of population, (b) population average density (number of person/hectare), (c) population density (number of person/hectare), (d) percent of population age < 16 years, (e) percent of population age 16–34 years, (f) percent of population age 35–64 years, (g) percent of population age > 64 years and (h) Parish wise area (%) and population (%).

The total population and average density of St Helens Deanery was 161,001 persons and 12.91 persons/ha (Table 3). Corpus Christi Parish (Id 1, Code 85) occupies the largest area of 2453.84 ha, 19.67% of St Helens Deanery but shares only 4.77% of the population with the lowest density of 3.13 person/ha (Table 3; Figures 6a,b,h). On the other hand, St Teresa Parish (Id 5, Code 144) shares 4.8% of the population with the highest density of 43.34 persons/ha (Table 3).

**Table 3:** Parish-wise area and population density at St Helens Deanery.

Id	Parish code	Parish name	Area (ha)		Population		
			Total	%	Total	%	Density (person/ha)
1	85	Corpus Christi	2453.84	19.67	7,673	4.77	3.13
2	3	St Mary	1414.31	11.34	7,158	4.45	5.06
3	113	St Thomas of Canterbury	953.61	7.64	14,734	9.15	15.45
4	206	St Julies	785.35	6.30	7,138	4.43	9.09
5	144	St Teresa	178.17	1.43	7,721	4.80	43.34
6	118	St Austin	611.46	4.90	15,684	9.74	25.65
7	64	Holy Cross	149	1.19	3,752	2.33	25.18
8	211	St Patrick	334.61	2.68	5,173	3.21	15.46
9	169	SS Peter and Paul	117.44	0.94	2,630	1.63	22.39
10	6	St Mary Immaculate	542.87	4.35	12,738	7.91	23.46
11	94	Blessed English Martyrs	735.09	5.89	10,814	6.72	14.71
12	63	St Mary and St John	1178.23	9.44	21,117	13.12	17.92
13	58	St Anne & Blessed Dominic	1030.97	8.26	23,792	14.78	23.08
14	139	St Teresa of the Child Jesus	1367.75	10.96	11,693	7.26	8.55
15	45	St Bartholomew	622.93	4.99	9,184	5.70	14.74
Total			12475.63	100	161,001	100	12.91

St Mary Immaculate (Id 10, Code 6) and St Anne & Blessed Dominic (Id 13, Code 58) hold high variation of density within the parish level (Figure 6c). According to age structure, a young population (age groups <16 years and 16–34 years) live in the central part of the deanery, dominated in St Austin (Id 6, Code 118), St Anne & Blessed Dominic (Id 13, Code 58), and Holy Cross Parishes (Id 7, Code 64) (Figures 6d,e). However, aged groups 35–64 years and > 64 years found in the north-western parts; Corpus Christi (Id 1, Code 85), St Mary (Id 2, Code 3), St Thomas of Canterbury (Id 3, Code 113) and partly in south and eastern part of the deanery St Mary and St John (Id 12, Code 63), St Teresa of the Child Jesus (Id 14, Code 139) Parishes (Figures 6f,g).

### 4.2 Hot spot/clustering

#### 4.2.1 Long-term health condition

**Figure 7:**
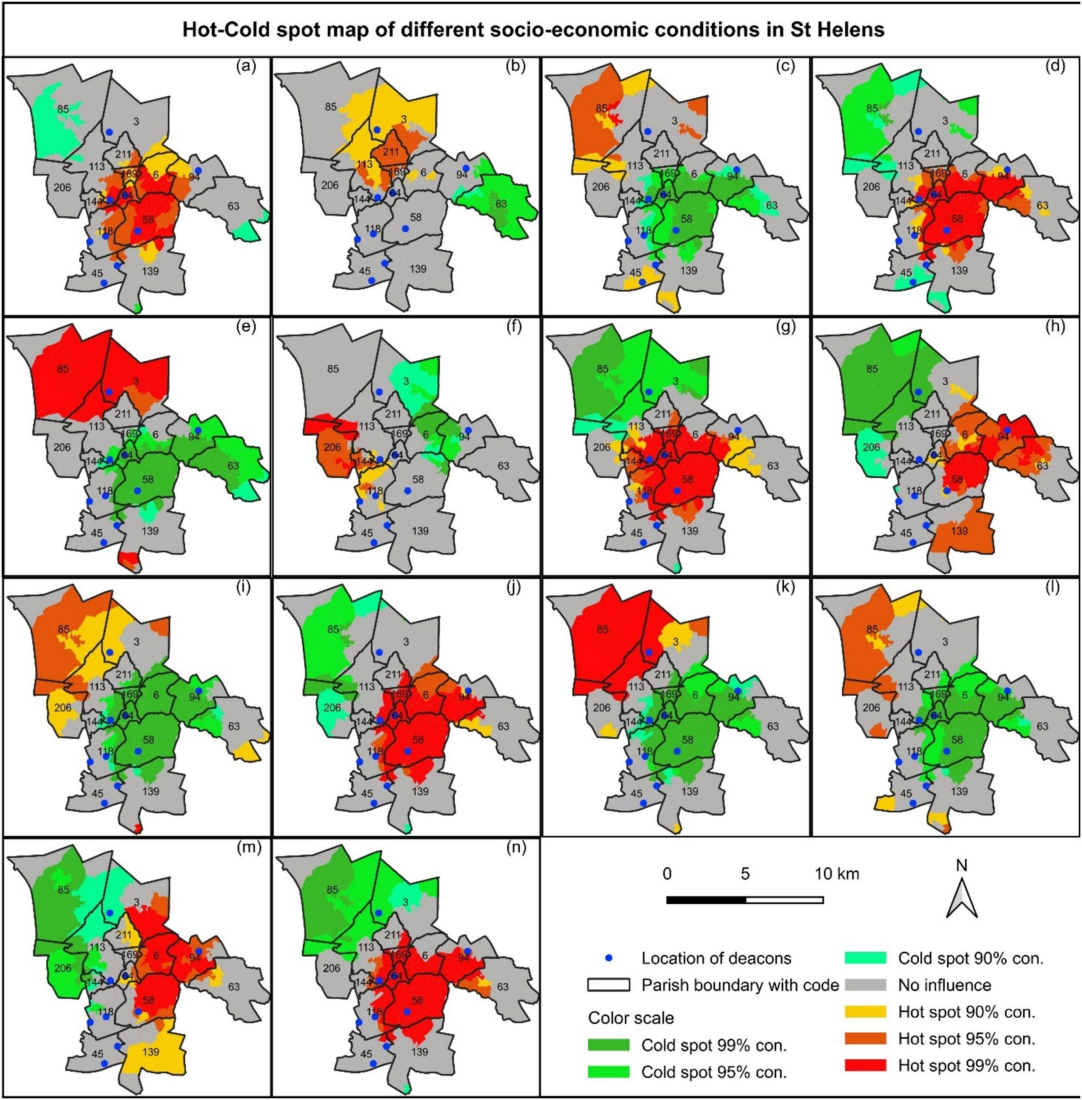
Hot-Cold spot map for long-term health conditions; (a) limited with a lot, (b) limited with little, tenure system; (c) owned, (d) rental house, economic situations; (e) retired, (f) students, (g) unemployed, education level; (h) level 1–3 and others, (i) level 4 and above, (j) no qualification, and social grade; (k) grade 1, (l) grade 2, (m) grade 3, and (n) grade 4 in St Helens.

Long-term health conditions limited the lifestyle of a lot of the population found in the central part of the deanery, in Holy Cross (Id 6, Code 64), St Anne & Blessed Dominic (Id 13, Code 58) and St Mary Immaculate Parishes (Id 10, Code 6) with the 95 to 99% confidence level (Figure 7a). On the other hand, long-term health conditions are rare in the Corpus Christi (Id 1, Code 85), and St Mary and St John (Id 12, Code 63) (Figure 7b), exposed in the cold spot with a 90% confidence level. However, St Mary (Id 2, Code 4), St Julies (Id 3, Code 113), and St Bartholomew (Id 45, Code 45) have no influence (Figure 7a). Among the parishes in St Helens Deanery, St Patrick (Id 8, Code 211) is exposed in the hot spot with 95% confidence level, and St Mary and St John (Id 12, Code 63) is exposed in the cold spot with the 99 and 95% confidence levels (Figure 7b). However, many parishes do not influence the term of long-term health condition lifestyle limited with little. Note that different parishes show partial results with various confidence levels. It was because the smaller geographic unit (civil boundary) was used as input.

#### 4.2.2 House ownership

The northern area of St Helens Deanery, Corpus Christi Parish (Id 1, Code 85) is exposed in the hot and cold spots with 95% confidence levels regarding owned and rental housing systems, respectively (Figures 6c,d). On the other hand, central parts of the deanery, St Anne & Blessed Dominic (Id 13, Code 58), Holy Cross (Id 7, Code 64), SS Peter and Paul (Id 9, Code 169), Blessed English Martyrs (Id 11, Code 94), and partially St Mary Immaculate (Id 10, Code 6) Parishes are exposed in the cold and hot spots with 95–99% confidence levels regarding owned and rental housing system, respectively (Figures 7c,d). The rest of the parishes have almost no influence.

#### 4.2.3 Economic factor

The northern part of St Helens Deanery, Corpus Christi (Id 1, Code 85) and St Mary (Id 2, Code 3) Parishes are dominated by exposing to hot spot with 99% confidence levels of retired people (Figure 7e). Regarding students, St Julies (Id 4, Code 206), and St Teresa (Id 5, Code 144) is exposed to hot spots with 95% confidence levels (Figure 6f). However, the central part, St Anne & Blessed Dominic (Id 13, Code 58), St Austin (Id 6, Code 118), St Teresa (Id 5, Code 144), St Thomas of Canterbury (Id 3, Code 113), Holy Cross (Id 7, Code 64), SS Peter and Paul (Id 9, Code 169), St Mary Immaculate (Id 10, Code 6), and Blessed English Martyrs (Id 11, Code 94) Parishes are exposed in the hot spot with 95–99% confidence level dominated by unemployed people (Figure 7g).

#### 4.2.4 Education qualification of residents

Hot spots for highly qualified people (level 4 and above) were found in the northern area of the deanery, Corpus Christi Parish (Id 1, Code 85) (Figure 7i). The central and the central-east regions, St Anne & Blessed Dominic (Id 13, Code 58), Holy Cross (Id 7, Code 64), SS Peter and Paul (Id 9, Code 169), St Mary Immaculate (Id 10, Code 6), Blessed English Martyrs (Id 11, Code 94), St Mary and St John (Id 12, Code 63) are dominated by the hot spot of no- and low-qualified people with 95–99% confidence levels (Figures 7h,j). On the other hand, the central part is the cold zone of the highly qualified people (Figure 7i).

#### 4.2.5 Social grade

The northern part of the deanery, Corpus Christi Parish (Id 1, Code 85) is exposed in the hot spot of the high-class families of grades 1 and 2 with 95–99% confidence levels (Figure 7a). The central part of St Helens Denary, St Anne & Blessed Dominic (Id 13, Code 58), Holy Cross (Id 7, Code 64), SS Peter and Paul (Id 9, Code 169), St Mary Immaculate (Id 10, Code 6), and Blessed English Martyrs (Id 11, Code 94) Parishes is dominated by the hot spot of low social grades (3 and 4) with the 95–99% confidence levels (Figures 7m,n). On the other hand, some parishes, e.g., St Bartholomew (Id 15, Code 45), St Julies (Id 4, Code 206), St Teresa of the Child Jesus (Id 14, Code 139), and St Mary and St John (Id 12, Code 63) have almost no influence according to social status (Figures 7k,n).

### 4.3 Deprived area based on served area

The central part, St Anne & Blessed Dominic (Id 13, Code 58), St Austin (Id 6, Code 118), SS Peter and Paul (Id 9, Code 169), St Mary Immaculate (Id 10, Code 6), Blessed English Martyrs (Id 11, Code 94), and partly St Mary and St John (Id 12, Code 63) Parishes are the most deprived areas in St Helens Denary who need more deacons and deacons’ services (Figure 6). On the other hand, the northern part, St Bartholomew (Id 15, Code 45) has the least influence either of hot and cold spot according to mentions of five socio-economic indicators but holds two permanent deacons (Figure 7). Considering one, one and a half and 2 km served street network from the existing deacons’ location, the most deprived area is estimated to 2505.91 ha, 2069.19 ha and 1449.95 ha that is 21.67, 19.40 and 15.4%, respectively, of the total area (Table 4). The deprived area is dominated by long-term health conditions (Figure 7a), fewer owned houses but more rental houses (Figures 7c,d), unemployed population (Figure 7g), less high level of qualification but no qualification (Figure 6j), and less social grade 1 and 2 but social grade 3 and 4 (Figures 7k,n). This is also a dense area of young people (Figures 6a,b,d,e).

**Table 4:** Deprived area with different constraints (existing served area).

Deprived ranks	1 km constraints		1.5 km constraints		2 km constraints	
	Area (ha)	%	Area (ha)	%	Area (ha)	%
1, Least deprived	894.08	7.73	894.08	8.38	894.08	9.50
2	2374.75	20.54	2342.93	21.97	2320.57	24.65
3	3451.68	29.85	3336.16	31.29	3157.23	33.54
4	2335.33	20.20	2021.30	18.96	1591.57	16.91
5, Most deprived	2505.91	21.67	2069.19	19.40	1449.95	15.40
Total	11561.74	100.00	10663.65	100.00	9413.39	100.00

## 5 Discussion

The study proposed an evidence-based approach to deploying permanent deacons using open and accessible government datasets. Applying an evidence-based approach is unique research in Roman Catholic communities in the deployment of permanent deacons. The UK is well advanced in open data publication; however, it is still challenging to analyze the multilevel spatial phenomena considering factors such as - population density, long-term health conditions, housing ownership, economic and social grade.

In the UK, particularly in the Archdiocese of Liverpool, parish-level population information (e.g., number, density) was not available. Estimation of parish-specific population and density (Figure 6; Table 3) in St Helens Deanery from the population in the civil boundary unit was a great success. Further, it was used as a strong factor in the MCA process (Table 2). Using parish-level population information and comparing it with the existing number of deacons and their location, the authority of the Archdiocese can easily gain insight into the inequality of deacon distribution, and, therefore, inequality of service allocations was expected. It may help the archdiocese in decision-making in better research allocation.

Hot spot analysis was conducted to study the spatial inequalities of socio-economic factors. Getis-Ord Gi* (ESRI, 2023) was applied for the identification of spatial inequalities (autocorrelation), which are objectively derived from attribute values (data). It is superior because of its ability to distinguish local event clusters of high and low features as suggested by Braithwaite and Li (2007) and Alam and Tabassum (2023), which is widely applied in the research communities in a similar context (e.g., Hassan et al., 2022; Roy et al., 2024c). Alam and Tabassum (2023). The spatial ranks from coldest to hottest cluster are generated along with statistics that clearly understand the need for deacons and their essential services in a spatial context (see sections 3.3 and 4.2).

MCA has been a widely used technique to identify suitable and optimal locations for service provision (e.g., Ampofo et al., 2023; Gamboa et al., 2023; Moisa et al., 2022; Zhou et al., 2022). MCA was applied to identify the deprived area where a permanent deacon or deacon’s services were most needed. Systematic analysis (evidence-based) for deploying permanent deacons is a unique study. It also allows human intervention through weighting the criteria, which could have both positive (e.g., applying weight based on local knowledge) and negative (e.g., considered a biased weighting) impacts during the evaluation process. This study involved weight values based on the opinion of experts who have complete knowledge about the local socio-economic conditions and internal as well as external factors of deployment of permanent deacons in the study area. However, this process ensures stakeholder participation in policy-making (e.g., Falanga and Nunes, 2021).

**Figure 8:**
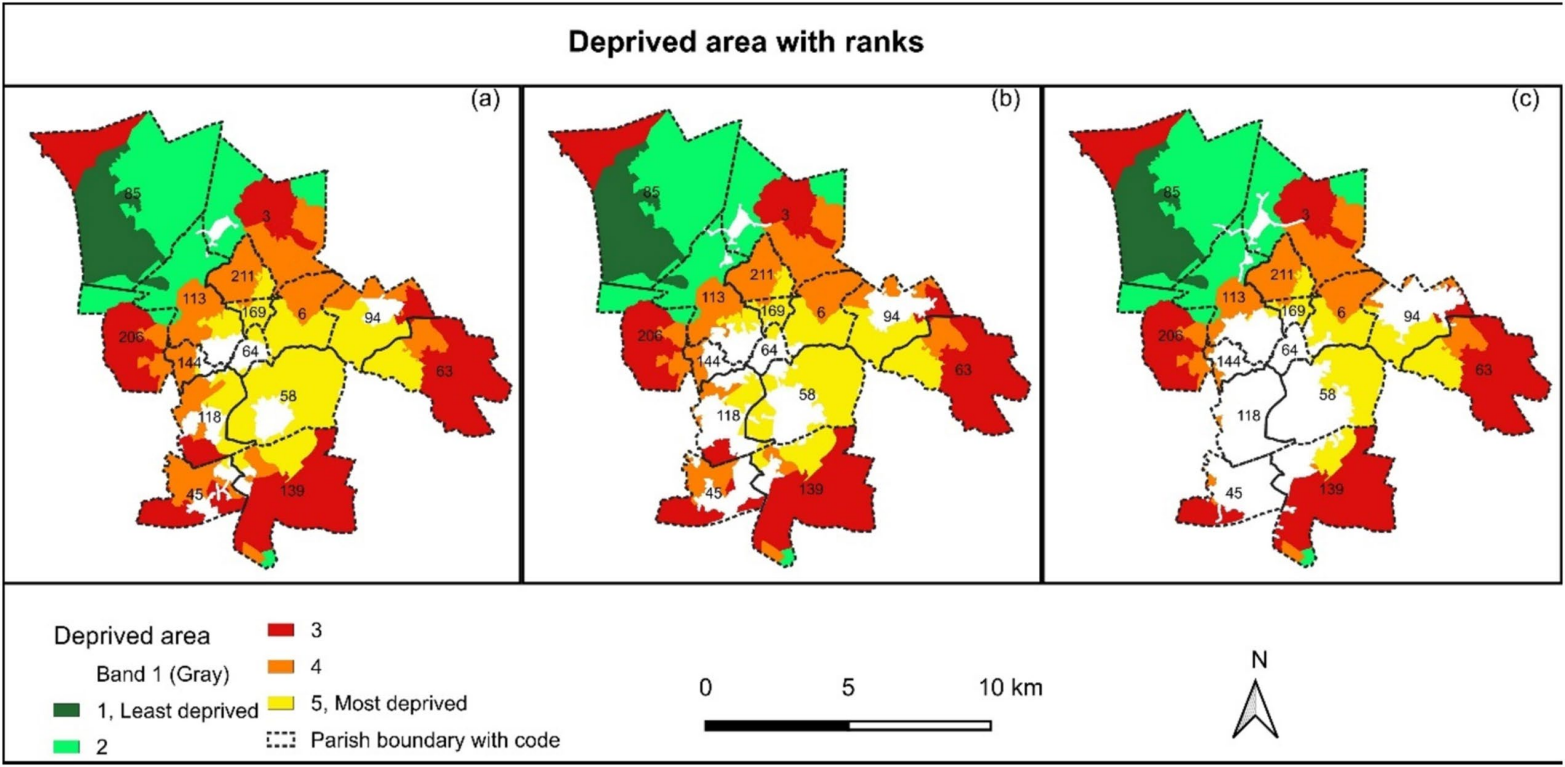
Deprived areas in the study area considering (a) 1-km, (b) 1.5-km, and (c) 2-km served road network area by the existing deacons.

The geographical unit problems were one of the significant challenges for the parish-wise direct comparison of inequality in the St Helens Denary was a challenge. So far, no studies have been found on socioeconomic disparities in parish-specific geographical units. The central part of the deanery is dominated according to the case study results in terms of the long-term health condition (Figure 7a), low ownership of a house and rental housing system (Figures 7c,d), high unemployment (Figure 7g), high non-qualified people (Figure 7j), families with low social grade (grade 3–4) (Figures 7m,n). Other studies also suggested that the central part is the most deprived area in terms of life expectancy, health condition and disability, education and skill, high suicide rate, under-18 conceptions, adult obesity, alcohol misuse and smokers, and diabetes register (e.g., St Helens Borough Council-SHBC, 2022; St Helens Public Health Team, 2020). Multiple deprivations in 2019 dominated the central part of the deanery (St Helens Borough Council-SHBC, 2022), which is equally found in our study even though this case study only could use the old census data from 2011 (Figure 8). On the other hand, the central part is the densest area, dominated by children (up to 16 y) and young adults (16–34 y) (Figure 6).

The interrelated factors are the level of education, social grade, income, house ownership, and long-term health conditions. Inequities in accessing essential services, e.g., healthcare, and education, also significantly contribute to socio-spatial disparities (Swope and Hernández, 2019). Policymakers can address these disparities by investing in underserved areas, enhancing and ensuring an equitable distribution of public services, thereby guaranteeing equal access to essential resources for all communities (Ravallion, 2001), which is equally suggested by St Helens Public Health Team (2020).

However, permanent deacons are appointed from the local communities and support their parish priest in religious activities. Besides, they engage in the life of their parish, and work to serve and exercise some leadership roles within the community, e.g., operating a food bank, involvement, and facilitating the education system (Diocese of Westminster-DoW, 2023). Among many, they can play an active role in sustainable development goals (SDGs); SDG 1: no poverty, SDG 2: zero hunger, SDG 3: good health and wellbeing, SDG 4: quality education, SDG 11: sustainable cities and communities (UNDP, 2024). Therefore, the appropriate number of permanent deacons from appropriate locations of deprived communities (as in Figure 8) can be directly involved in resolving socio-economic inequalities on a spatial scale.

This study was based on freely available open-source data. Combining multi-dimensional open-source data sets is challenging for policy-making considering spatial–temporal resolutions (e.g., Franklinos et al., 2020; Islam et al., 2023). The uncertainty in estimating such open-source data might be an issue, e.g., parish-wise population provides a close approximation estimated from density (section results: population and density, Table 3). Estimating the ranks of deprived areas is expected not to be affected by the data issue in this study. However, the results of this study reflect the reality that satisfied the expectations of the local stakeholders. This statement was confirmed by cross-checking the comments from a participant who attended the workshop and said, “this is what we see in our deanery.”

The study gathers the best available dataset for assessing the local resource distribution and local leadership. The spatial equality has been addressed by applying an established spatial algorithm. This study provides statistical attributes and visual maps, which should be helpful for geovisual communication with the concerned stakeholders in deacon deployment. Transparency in decision-making will also be improved if the proposed approach has been adopted after being applied to more empirical case studies, even in other geographical contexts. However, the data harmonization method has been applied to harmonize the input dataset, which can pose some uncertainty. Therefore, expert opinion-based validation of the results is relevant for reducing some degree of confidence in the empirical results.

Note that several limitations exist in this study. (i) One such limitation is the limited data used in this study to explore spatial-socioeconomic inequality. For example, household income and life expectancy at birth were significant factors in decision-making to identify needs for permanent deacons or deacons’ services, which were not used in this study. However, it is a start to conduct such a study and provides a pipeline where more data layers can be filled in. A new data set from the Ordnance Survey, 2021 might offer more insight into the conditions. (ii) Constraints were estimated considering a certain distance along the road network, which did not consider the density, which might be another issue. (iii) Weighting might be a bias depending on how the researchers see the influence of factors in the decision-making process (Heywood et al., 2011, p. 247). In this study, the weighting was expected to be judged, derived from expert respondents who were full of knowledge in both context and content. However, specific interview respondents (e.g., students, unemployed, rental house owners) from the local area (St Helens) with sufficient samples might have different opinions and thus different results. Further study might consider these issues to investigate the scenarios.

## 6 Conclusion

Investigation of the evidence-based allocation and deployment of permanent deacons was a challenge due to the different geographical unit (parish) of the St Helens Deanery of the Archdiocese of Liverpool and data used based on political boundaries (Figure 2). However, this study explored the scenarios that were in line with others, particularly the inventory of the St Helens Council. Socio-economic inequality and gross and age-specific density suggest that the central part (St Anne & Blessed Dominic, Holy Cross, SS Peter and Paul, St Teresa, Blessed English Martyrs, St Mary Immaculate Parishes) is the most deprived area. The study also provides the rank of the depravedness of the study area, which can be taken into consideration by the Archdiocese of Liverpool in the deployment of permanent deacons to meet the needs of the people. However, most importantly, this study offers the methodology of how socio-economic factors can be considered in resource allocation in the religious community which makes this study unique.

However, for further research, the following issues may be considered for the deployment of permanent deacons and allocation of related resources.

(a) The whole analytical procedure may be conducted using the parish-level spatial and aspatial data with better temporal and spatial resolution which may provide more accurate results.
(b) The qualitative and quantitative, hybrid methods may be applied which may consider social perceptions and experiences in inequality studies.
(c) More socio-economic factors need to be incorporated for a better understanding of the insight the society.
(d) Causal relationships among the factors may exist which may be explored and incorporated in the models to describe inequality.
(e) If stakeholders need to be involved, a sufficient number/ statistically significant number of them could be considered to acquire a better reflection from society.

## Data Availability

Publicly available datasets were analyzed in this study. This data can be found at: https://digimap.edina.ac.uk/os.

## Footnotes

^1^ https://euare2023.wp.st-andrews.ac.uk/
