# Supplementary material for: Sensing spatial inequality of socio-economic factors for deploying permanent deacons in the UK
Source: Front Sociol. 2024 Nov 1;9:1481413. doi: 10.3389/fsoc.2024.1481413 (PMC11565288; doi:10.3389/fsoc.2024.1481413)
Supplement: Supplementary file 1 [file Data_Sheet_1.docx]

**Appendix A**

**
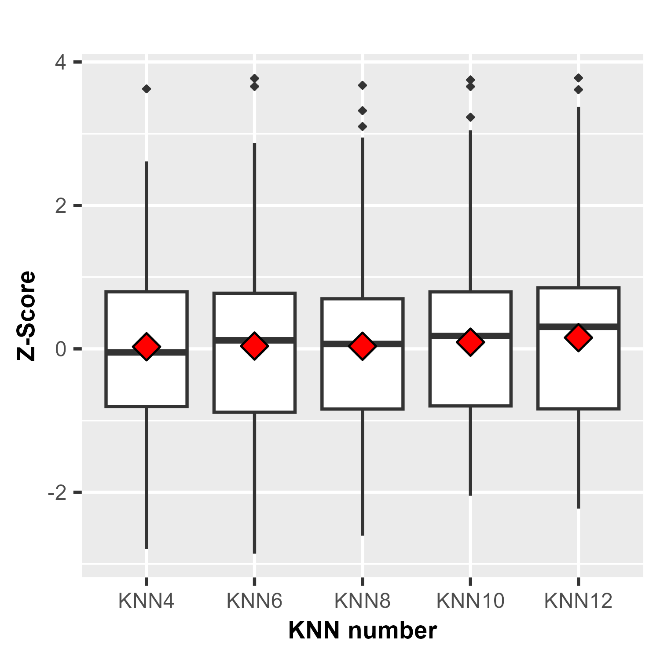
**

**FIGURE A1** Z-score distribution of K Nearest Neighbors (KNN) in Getis-Ord Gi* clustering for long-term health conditions limited with lot.

**
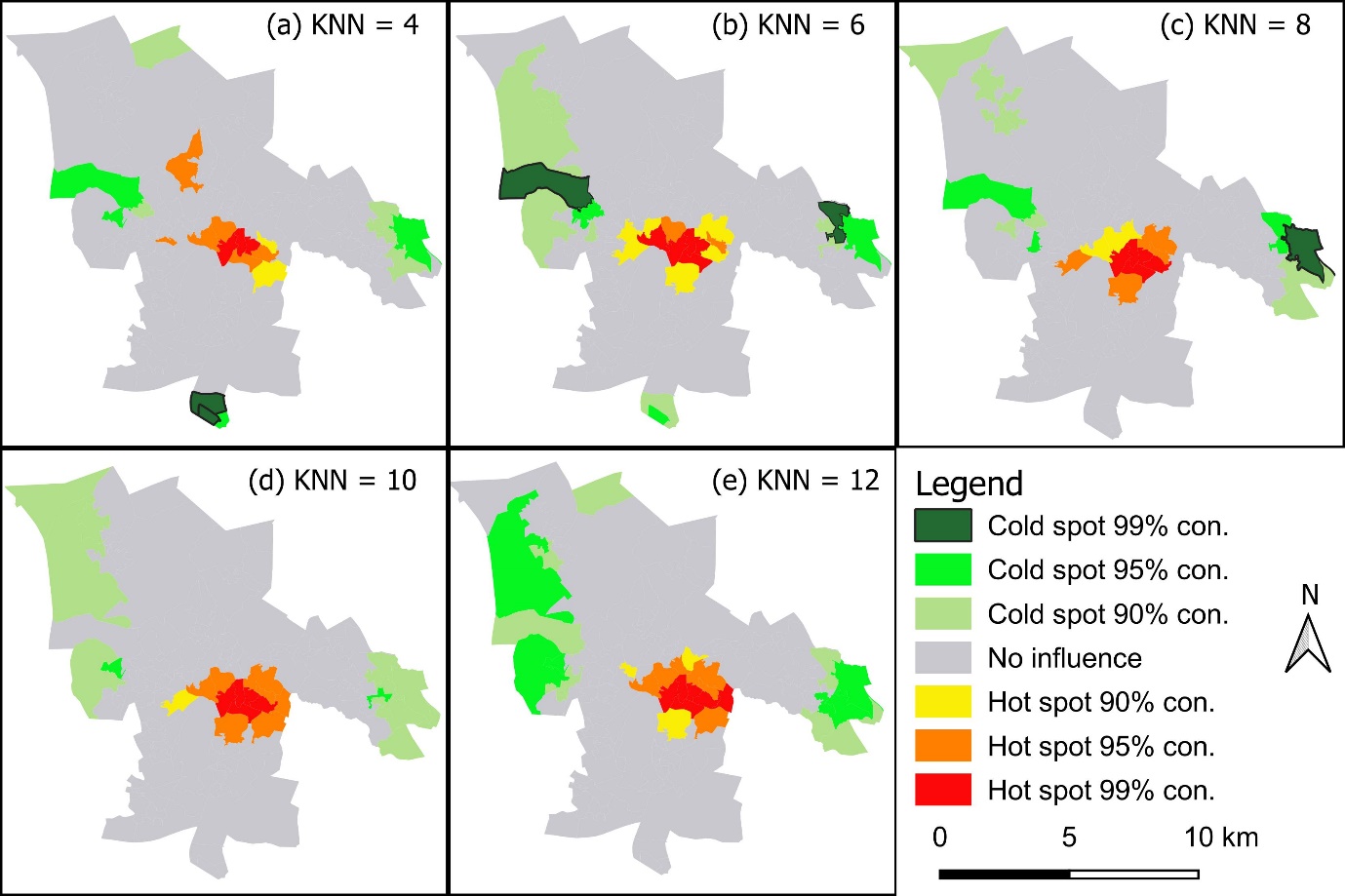
**

**FIGURE A2** Hot spots region with different numbers of K Nearest Neighbors (KNN) in Getis-Ord Gi* clustering **for** long-term health conditions limited with lot.

**Appendix B**

**Questionnaire: Sensitivity of socio-economic parameters in the deployment of Roman Catholic permanent deacons.**

**Long Term Health condition**

Those suffering from long-term health conditions need more deacons or deacons’ services (please tick or cross here).

| Strongly agreed | Agreed | Fairly agreed | Disagree | Strongly disagree |
| --- | --- | --- | --- | --- |
|  |  |  |  |  |

**Employment**

Unemployed people need more deacons or deacons’ services (please tick or cross here).

| Strongly agreed | Agreed | Fairly agreed | Disagree | Strongly disagree |
| --- | --- | --- | --- | --- |
|  |  |  |  |  |

Retired people need more deacons or deacons’ services (please tick or cross here).

| Strongly agreed | Agreed | Fairly agreed | Disagree | Strongly disagree |
| --- | --- | --- | --- | --- |
|  |  |  |  |  |

Students need more deacons or deacons’ services (please tick or cross here).

| Strongly agreed | Agreed | Fairly agreed | Disagree | Strongly disagree |
| --- | --- | --- | --- | --- |
|  |  |  |  |  |

**Tenure system**

Those with owned tenure condition need more deacons or deacons’ services (please tick or cross here).

| Strongly agreed | Agreed | Fairly agreed | Disagree | Strongly disagree |
| --- | --- | --- | --- | --- |
|  |  |  |  |  |

Those with rental tenure conditions need more deacons or deacons’ services (please tick or cross here).

| Strongly agreed | Agreed | Fairly agreed | Disagree | Strongly disagree |
| --- | --- | --- | --- | --- |
|  |  |  |  |  |

**Qualification**

People with education levels 1-3 and others (A level, O level, work-related education) need more deacons or deacons’ services (please tick or cross here).

| Strongly agreed | Agreed | Fairly agreed | Disagree | Strongly disagree |
| --- | --- | --- | --- | --- |
|  |  |  |  |  |

People with education level 4 and above (bachelor, Master, Ph.D., etc.) need more deacons or deacons’ services (please tick or cross here).

| Strongly agreed | Agreed | Fairly agreed | Disagree | Strongly disagree |
| --- | --- | --- | --- | --- |
|  |  |  |  |  |

People without qualifications need more deacons or deacons’ services (please tick or cross here).

| Strongly agreed | Agreed | Fairly agreed | Disagree | Strongly disagree |
| --- | --- | --- | --- | --- |
|  |  |  |  |  |

**Social grade**

People with social grade 1 need more deacons or deacons’ services (please tick or cross here).

| Strongly agreed | Agreed | Fairly agreed | Disagree | Strongly disagree |
| --- | --- | --- | --- | --- |
|  |  |  |  |  |

People with social grade 2 need more deacons or deacons’ services (please tick or cross here).

| Strongly agreed | Agreed | Fairly agreed | Disagree | Strongly disagree |
| --- | --- | --- | --- | --- |
|  |  |  |  |  |

People with social grade 3 need more deacons or deacons’ services (please tick or cross here).

| Strongly agreed | Agreed | Fairly agreed | Disagree | Strongly disagree |
| --- | --- | --- | --- | --- |
|  |  |  |  |  |

People with social grade 4 need more deacons or deacons’ services (please tic or cross here).

| Strongly agreed | Agreed | Fairly agreed | Disagree | Strongly disagree |
| --- | --- | --- | --- | --- |
|  |  |  |  |  |

Thanks for your time and contribution!
